# Tamm-Horsfall protein facilitates catheter associated urinary tract infection

**DOI:** 10.1186/1756-0500-5-532

**Published:** 2012-09-26

**Authors:** Hajamohideen S Raffi, James M Bates, Dayl J Flournoy, Satish Kumar

**Affiliations:** 1 Department of Medicine/Nephrology, University of Oklahoma Health Sciences Center and Veterans Affairs Medical Center, 920 SL Young Blvd., WP 2250, Oklahoma City, OK 73104, USA; 2 Department of Pathology, University of Oklahoma Health Sciences Center and Veterans Affairs Medical Center, Oklahoma City, OK 73104, USA

**Keywords:** Tamm-Horsfall protein, Urinary catheters, Bacterial binding

## Abstract

**Background:**

Urinary catheters are associated, commonly with bacteriuria and frequently with urinary tract infection. Tamm-Horsfall Protein (THP) is urine's most abundant protein and is known to bind to uropathogenic bacteria. The role of THP in the pathogenesis of catheter associated urinary tract infection (CAUTI) is not clear. We examined the role of THP in facilitating bacterial binding to urinary catheters *in vivo* and *in vitro*.

**Findings:**

Twenty one urinary catheters were obtained from 20 hospitalized patients. THP was eluted from the catheter surface and catheter segments were cultured. Additional studies were performed *in vitro* on unused silicone and latex catheters to determine the binding of THP, and the effect of THP on the binding of *Escherichia coli* (*E. coli*) and *Pseudomonas aeruginosa* (*P. aeruginosa*), to the catheter surface.

On catheters obtained from patients, the THP deposition was significantly more on culture positive catheters than on culture negative catheters. In the *in vitro* studies, THP bound to both silicone and latex catheters, and THP enhanced the adherence of *E. coli and P. aeruginosa* to both types of catheters.

**Conclusion:**

THP binds to urinary catheters and facilitates the binding of uropathogenic bacteria to catheters.

## Background

Catheter Associated Urinary Tract Infection (CAUTI) is a common hospital acquired infection. Urinary catheters are placed in one of four hospitalized patients in the United States [1]. About 26% of catheterized patients develop asymptomatic catheter associated bacteriuria (CAB) of which 24% progress to symptomatic CAUTI [2]. CAUTI accounts for 40% of all hospital acquired infections [3,4] and 80% of all hospital acquired urinary tract infections (UTI) [5]. In adult intensive care units, more than 95% of urinary tract infections are caused by urinary catheters [6].

Tamm-Horsfall protein (THP) is the most abundant protein in normal urine with multiple postulated biological functions [7-9]. THP is a glycoprotein with a variety of n-linked and o-linked glycans that give it a versatile ability to bind a variety of substances [7,8]. We [10] and others [11] have previously shown that THP plays a defensive role against UTI presumably by binding uropathogenic bacteria and helping to clear them from the urinary tract.

The role of THP as a host defense factor against UTI has not been studied in the presence of an in-dwelling urinary catheter. The abundance of THP in urine and its tendency to bind a variety of surfaces make it likely that it would bind to the surface of urinary catheters. We hypothesized that the presence of a catheter in the urinary tract may reverse the normal role of THP. Normally -- in the absence of a catheter -- THP acts as a host defense factor against UTI. The presence of a catheter, however, could allow THP to bind to the catheter surface and form a conditioning layer that could allow binding of bacteria and formation of a bacterial biofilm. Bacteria could multiply undisturbed in the biofilm [12] and be released into the urine upon reaching an adequate density, perpetuating bacterial presence in the urine.

At present, it is not known if THP sticks to urinary catheters and if it affects the binding of bacteria to the catheter surface. In the present study, we determined the effect of THP on binding of uropathogenic bacteria *in vivo* and *in vitro* to commonly used urinary catheters in the United States. Urinary catheters collected from patients were examined for THP and bacterial adherence. In the *in vitro* studies, silicone and latex catheters were examined for adsorption of THP and the effect of THP on the adherence of two types of uropathogenic bacteria to the catheter surface.

## Methods

### Human studies

Twenty one urinary catheters (17 latex and 4 silicone) were collected aseptically from 20 patients after approval from the institutional review board of The University of Oklahoma Health Sciences Center, Oklahoma City, OK. The catheter insertion and removal was performed as considered necessary for the routine clinical care by the treating physicians. The catheters were cut into 1 cm segments starting at the catheter tip. One segment was used for quantitation of THP binding and another for bacterial culture. Bacteria were dislodged from the catheter section by sonication and cultured on blood and MacConkey agars. Bacterial isolates were speciated with the Vitek 2 System (bioMerieux, Inc., Durham, NC) using colorimetric technology. THP was eluted from catheter segments by incubation in TEA buffer (0.5% Triton-X 100/20 mM EDTA/pH 7.5) and quantitated by ELISA.

### In-vitro studies

Human urine was collected in a sterile container and tested for sterility on MacConkey agar. One half of the urine specimen was filtered through a 0.2 μ filtration unit (Nalgene, Rochester, NY) to remove THP. The whole and filtered urine specimens were analyzed for THP by ELISA and polyacrylamide gel electrophoresis followed by silver staining and Western Blot.

#### THP binding experiments

Bardex Infection Control (latex) and Lubri-Sil Infection Control (silicone), 20 French catheters were cut into 1-cm segments. The catheter segments were incubated in 40 ml of whole or filtered urine, in 50 ml sterile conical centrifuge tubes. The tubes were rotated at 37°C and the solutions were changed daily. Three catheter segments were removed at 9, 12 and 24 hrs and on days 7, 14 and 30, and rinsed in phosphate buffered saline (PBS). THP bound to the catheter segments was extracted in TEA buffer and quantitated by ELISA.

#### Bacterial adherence experiments

*Escherichia coli* (*E. coli*) strain UTI89 (a clinical cystitis strain mainly expressing type 1 fimbriae) and *Pseudomonas aeruginosa* (*P. aeruginosa*, American Type Culture Collection # 27314) were grown in Tryptose Broth and Brain Heart Infusion Broth, respectively. The concentration of *E.coli* in the broth was measured by culture of serial dilutions of the broth on Eosin Methylene Blue (EMB) agar and that of *P. aeruginosa* on Columbia Blood Base Agar. The bacteria were grown for 24 hours at 37°C with 5 uCi/ml of methyl-^3^H Thymidine (64 Ci/mmol specific activity). The radiolabeled bacteria were washed in Dulbecco's phosphate buffered saline (DPBS) and resuspended at a concentration of 1x10^8^ CFU/ml, in artificial urine [13] or artificial urine with 0.1 mg/ml THP. The bacterial solutions were incubated with 1-cm sections of the latex and silicone catheters at 37°C. Three sections were removed from each tube at 1, 6 and 24 hrs, and washed with DPBS. Bacterial binding to the catheter segments was measured by scintillation counting. On days 2, 4 and 7, three sections were removed from each tube, and sonicated in DPBS to dislodge the bacteria. The bacteria were quantitated by culture of serial dilutions on agar.

### Statistical analysis

The data were expressed as the mean + SE and compared statistically using Student’s t-test. Statistical significance was set at P < 0.05.

## Findings

### Human studies

**Figure 1:**
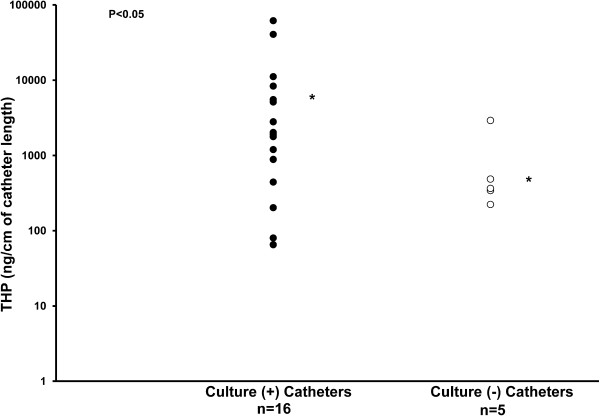
**Distribution of catheter THP concentration and catheter bacterial culture analysis (positive or negative).** The bacterial culture positive catheters had significantly more THP on the catheter surface. * indicates the mean.

Cultures of catheter segments were positive in 16 (15 latex and 1 silicone) and negative in 5 (2 latex and 3 silicone) catheters. The organisms isolated and the number of culture positive catheters were: *Escherichia coli*, 3; *Enterococcus fecium*, 3; *Enterococcus faecalis*, 5; *Staphylococcus epidermidis*, 4; *Candida albicans*, 1; *Staphylococcus hominis*, 1; *Klebsiella pneumoniae*, 1; *Staphylococcus aureus*, 1; *Enterobacter sakazakii,* 1 and *Staphylococcus warneri*, 1; some catheters had multiple organisms. The THP deposition was significantly more on culture positive catheters than on culture negative catheters (8990 ng THP/cm + 4301 vs. 865 ng THP/cm + 513, respectively; P = 0.039) (Figure 1).

### In-vitro studies

**Figure 2:**
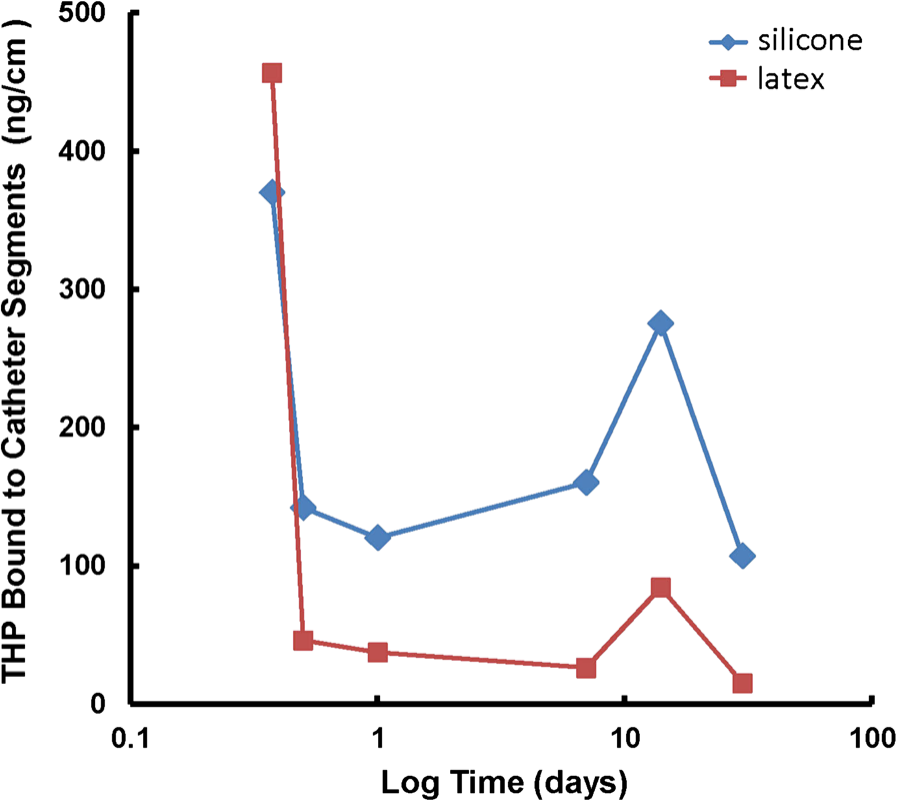
**Time Course of THP adsorption to latex and silicone urinary catheters.** THP adsorption increased on both types of catheters after 7 days. THP adsorption was more on silicone catheters.

Both silicone and latex urinary catheters were quickly and equally coated with THP from the first time point at 9 hrs (silicone, 370 ng/cm + 65 and latex, 456 ng/cm + 155). At later time points, more THP was found bound to the silicone catheters than to the latex catheters respectively: 12 hr (142 ng/cm + 15 vs 46 ng/cm + 37, P = 0.001), day 1 (120 ng/cm + 4 vs 37 ng/cm + 5, P = 0.00001), day 7 (160 ng/cm + 33 vs 26 + 5 ng/cm + , P = 0.003), day 14 (275 ng/cm + 41 vs 84 ng/cm + 4, P = 0.002) and day 30 (107 ng/cm + 32 vs 15 ng/cm + 5, P =0.014). THP binding to urinary catheters increased further after 7 days of incubation (Figure 2).

*E. coli* adherence (expressed as Log CFU/cm catheter), in the artificial urine with THP versus artificial urine without THP was greater to the silicone catheter at 1 hr (6.25 + 0.17 vs 5.73 + 0.07, P = 0.02), 6 hr (6.08 + 0.04 vs 5.67 + 0.13, P = 0.02), day 1 (6.1 + 0.02 vs 5.57 + 0.02, P = 0.00004) and day 7 (4.2 + 0.2 vs 2.9 + 0.2, P = 0.005) and to the latex catheter at 6 hr (5.95 + 0.06 vs 5.68 + 0.05, P = 0.01) than in artificial urine alone. *P. aeruginosa* adherence was more to the latex catheter at 2 days (6.7 + 0.03 vs 6.4 + 0.01, P = 0.0003), and to the silicone catheter at 7 days (4.0 + 0.2 vs 2.8 + 0.4, P = 0.03) in the artificial urine with THP versus the artificial urine alone.

## Discussion

The urinary tract is normally sterile. Insertion of a bladder catheter invades this sterile space and provides an opportunity for infection. *E.coli* accounts for about 25-50% of the cases of CAUTI. Other species isolated frequently are *Proteus, Klebsiella, Enterococcus, Pseudomonas* and *Staphylococcus*[14,15]. These bacteria are normally resident in the colon and frequently colonize the periurethral space. They ascend up the catheters into the urinary bladder on the external surface of the catheter in 66% and on the lumen of the catheter in 34% of the cases [16]. For CAUTI to occur, the bacteria need to adhere to the catheter, have an opportunity to multiply, evade normal urinary defense factors and invade the bladder epithelium.

THP has long been postulated to play a role in binding free floating (planktonic) bacteria in the urine and in aiding their excretion to help maintain the sterility of the urinary tract [17]. *In vitro* studies have demonstrated binding between *E. coli* and THP that was mediated by the binding of type 1 fimbriae on the surface of *E. coli* to the high-mannose structure present on THP [18]. The role of THP as a urinary defense factor was highlighted when we and others created THP gene knockout mice and demonstrated that THP-deficient mice had difficulty clearing *E. coli* from the urinary tract [10,11]. Subsequently, we extended these findings to include other uropathogenic bacteria such as *Klebsiella pneumonia, Staphylococcus saprophyticus*, [19] and *Proteus mirabilis*[20]. In a study examining 51 long term catheterized patients, the bacterial isolates from UTI episodes longer than 2 weeks expressed type 1 fimbriae more frequently and bound more THP than bacterial isolates from UTI episodes lasting 1 week or less [21]. These data suggested that binding between type 1 fimbriae and THP could have contributed to the persistence of CAUTI.

In our clinical study, all catheters were found to be coated with THP and the amount of THP adsorbed was higher on culture positive catheters. In our *in vitro* studies, THP bound to both latex and silicone urinary catheters and facilitated the binding of *E. coli* and *P. aeruginosa* to both types of catheters. THP binding to urinary catheters increased after 7 days.

These data suggest that THP which serves as a host defense factor against UTI in the absence of an indwelling urinary catheter may have the opposite effect of promoting bacterial presence in urine in the presence of a urinary catheter and that catheter removal or change at 7 days may help prevent CAUTI. The major limitation of this study is the small sample size. These data need to be confirmed in larger studies before routine change of catheters at 7 days can be recommended as a practice guideline.

## Conclusion

THP, a normal host defense factor against UTI, may have a paradoxical effect of promoting UTI in the presence of indwelling urinary catheters.

## Competing interests

The authors declare that we have no competing interests.

## Authors' contributions

All authors made substantial intellectual contribution. HSR was involved in coordination of human studies, collection of data, interpretation and reporting of data and drafted the manuscript. JMB was involved in performing *in vitro* studies, collection of data from human studies, statistical analysis and interpretation of data and helped to draft manuscript. DJF was involved in bacterial identification. SK was involved in conception, design and supervision of the experiments, interpretation of results, and in preparation of the final draft. All authors read and approved the final manuscript.
